# Здоровое долголетие - что может сделать гинеколог?

**DOI:** 10.14341/probl13644

**Published:** 2025-09-14

**Authors:** Е. Н. Андреева, Е. В. Шереметьева, О. Р. Григорян

**Affiliations:** Национальный медицинский исследовательский центр эндокринологии им. академика И.И. Дедова; Российский университет медицины; I.I. Dedov Endocrinology Research Centre; Russian University of Medicine; Национальный медицинский исследовательский центр эндокринологии им. академика И.И. Дедова; I.I. Dedov Endocrinology Research Centre

**Keywords:** менопауза, менопаузальная гормональная терапия, дидрогестерон, эстрадиол, саркопения, миома матки, menopause, menopausal hormone therapy, dydrogesterone, estradiol, sarcopenia, uterine fibroids

## Abstract

A woman spends more than a third of her life in a state of deficiency of female sex hormones. According to WHO, in most countries of the world the life expectancy of women after 50 years fluctuates between 27 and 32 years. Every year the number of women entering the menopause period increases. In 1990, 467 million were in the postmenopause period, by 2030 the number will reach 1.2 billion. Menopause, not being a disease itself, leads to a violation of the endocrine balance in the body, causing not only «classic» problems in life (vasomotor symptoms, psychological health disorders, urogenital disorders, osteoporosis, cardiovascular diseases), but also changes the appearance of women — the dermatological status of the patient is worse than the age group. The article presents modern data on rational MHT. Particular attention is paid to the issues of efficacy, tolerability and safety of combined MHT containing estrogen and gestagen, based on the results of current studies and in accordance with the position of national and international clinical guidelines. A clinical case is used to demonstrate the tactics of managing a woman in menopause.

## АКТУАЛЬНОСТЬ

В настоящее время медицинская общественность проявляет все больший интерес к актуальному междисциплинарному направлению — anti-age-медицине (медицине «антистарения»), целью которой является индивидуализация выявления инволюционных изменений организма и комплексный междисциплинарный подход при их коррекции. Современная демографическая ситуация характеризуется увеличением продолжительности жизни, а следовательно, и ростом популяции пожилых людей. В России количество женщин пери- и постменопаузального возраста составляет более 21 млн. Согласно мировым статистическим данным, средний возраст наступления менопаузы во всем мире составляет 48,8 года, с колебаниями этого показателя в зависимости от географического региона проживания женщин, в РФ он колеблется от 49 до 51 года. К 2030 г., по демографическим прогнозам, более 1,2 млрд вступят в период менопаузы [3][4]. Приверженность к терапии как важный аспект применения МГТ и индивидуальный подход стали приоритетными направлениями при выборе тактики лечения климактерических расстройств. Неотъемлемой частью данной концепции являются: определение лекарственной формы препаратов МГТ, подбор минимальной эффективной дозировки, использование режима терапии с учетом возраста, стадии репродуктивного старения (STRAW +10) и потребности пациентки [5].

## ОПИСАНИЕ СЛУЧАЯ

Пациентка Л. (1958 г.р.) обратилась в отделение эндокринной гинекологии ГНЦ РФ «ФГБУ НМИЦ эндокринологии» МЗ РФ впервые в декабре 2003 г. в возрасте 45 лет с жалобами на обильные менструации в течение последнего года. По данным УЗИ органов малого таза, было выявлено образование правого яичника (фолликулярная киста правого яичника), которое периодически пациентка наблюдала с 2001 г. Проводилась неоднократно терапия препаратом дидрогестерон (20 мг в сутки) во II фазу цикла с регулярной менструало-подобной реакцией. В марте 2003 г. проведено РДВ по поводу полипа цервикального канала, по данным гистологического заключения — фиброзно-железистый полип. По данным УЗИ органов малого таза, в октябре 2003 г. аденомиоз, множественная миома матки небольших размеров, рецидив образования правого яичника (фолликулярная киста) 4 см в диаметре. Был повторно рекомендован курс приема дидрогестерона по 1 таблетке 2 раза в день по II фазе менструального цикла, менструалоподобная реакция регулярная. Далее в течение трех лет пациентка ежегодно проводила плановые гинекологические обследования, патологии выявлено не было. В 2006 г. в возрасте 47 лет женщина вновь обратилась в отделение эндокринной гинекологии с жалобами на нарушения менструального цикла по типу задержек до 50 дней в течение последнего года, усиление потливости и чувства жара днем даже без физической нагрузки, которое еще более усиливается на фоне «задержек» менструации; прибавку массы тела за последний год на 5 кг (в н.м. вес 70 кг) на фоне неизменного питания и образа жизни, а также перепады настроения. У пациентки 3 беременности, двое родов (естественные роды, вес детей 3,2 и 3,5 кг, гестационный сахарный диабет отрицает, грудное вскармливание до 1 года), одно искусственное прерывание беременности, половая жизнь регулярная, метод предохранения — барьерный. Из сопутствующих заболеваний следует отметить эпизодическое повышение АД не более 140 мм рт.ст. на фоне стрессовых ситуаций, которое участилось в последний год. Наследственный анамнез отягощен по сахарному диабету 2 типа (СД2) по материнской линии. ИМТ — 25,2 кг/м², ОТ — 89 см, АД на правой руке — 118/78 мм рт.ст., на левой руке — 121/80 мм рт.ст., ЧСС — 76 уд/мин. По данным гинекологического осмотра наружные половые органы развиты правильно, оволосение по женскому типу, шейка матки при осмотре в гинекологических зеркалах — без особенностей, матка увеличена до 6 недель условной беременности, неоднородная, бугристая, подвижная, пальпация безболезненная, придатки не пальпируются, область пальпации безболезненная. Выделения из половых путей слизистые, без запаха.

## Данные лабораторных и инструментальных методов исследования

По данным маммографии выявлена доброкачественная дисплазия молочных желез, BIRADS 2, по результатам УЗИ малого таза — эхографические признаки аденомиоза, множественные узлы субсерозного расположения, max 2,5 см в диаметре, протокол УЗИ органов брюшной полости и почек показал диффузные изменения печени по типу жирового гепатоза, диффузные изменения поджелудочной железы (липоматоз), по данным ЭКГ и УЗИ щитовидной железы значимых изменений не выявлено. Биохимический анализ крови: общий белок — 71 г/л, АЛТ — 25 Ед/л (норма до 33), АСТ — 18 ЕД/л (норма до 32), ГГТ — 25 Ед/д (до 42), креатинин — 78 мкмоль/л, мочевина — 4,7 ммоль/л, мочевая кислота — 340 ммкмоль/л (150–350), общий холестерин — 5,9 ммоль/л, ЛПНП — 3,39 ммоль/л, ЛПВП — 1,1 ммоль/л, ТГ — 2,1 ммоль/л, 25-ОН-D — 43 нг/мл (пациентка ежедневно принимает 2000 МЕ колекальциферола), гормональный анализ крови: ТТГ — 1,2 мЕд/мл, ФСГ — 34 Ед/л, при повторном измерении — 17 Ед/л, ЛГ — 15 Ед/л, эстрадиол — 121 пмоль/л, пролактин — 125 мЕд/мл. Мазок на жидкостную онкоцитологию — NILM. Коагулограмма — нормокоагуляция. Клинический диагноз пациентки 47 лет, обратившейся в отделение эндокринной гинекологии: «Климактерический синдром. Период менопаузального перехода. Аденомиоз (по данным УЗИ). Миома матки с субсерозным расположением узла. Дислипидемия. Неалкогольная жировая болезнь печени. Избыточная масса тела (ИМТ — 25,2 кг/м²)». Пациентка отказалась от приема МГТ, опасаясь возможных побочных реакцией, поэтому ей было рекомендовано: негормональная коррекция вазомоторной симптоматики (чувства жара и потливость), согласно клиническим рекомендациям «Менопауза и климактерическое состояние» и международным протоколам [5][10], дидрогестерон 10 мг 1 таблетка 2 раза в день после еды с 16-го по 25-й дни менструального цикла — 6 месяцев, а также консультация диетолога и психолога/психотерапевта в НМИЦ эндокринологии, через 6 месяцев явка на консультацию для оценки клинического эффекта (в плане — инициация МГТ).

Через 6 месяцев на повторной консультации пациентка отметила, что на фоне приема дидрогестерона нет регулярной менструалоподобной реакции (МПР) или она очень скудная темно-коричневыми выделениями, прибавка массы тела продолжается, еще +4 кг за 6 мес (не обратилась на консультацию к диетологу и психоневрологу: «думала, справлюсь сама»), постоянную потливость, ночные поты 3–4 раза за ночь, тревожный, прерывистый сон, выраженные отеки утром. После очередной разъяснительной беседы пациентка согласилась принимать эстрадиол/дидрогестерон (1 мг/10 мг) по 1 таблетке 1 раз в день в одно и то же время суток без перерыва, пачка за пачкой, на последних таблетках 3-й пачки провести УЗИ малого таза, а также обратиться на консультацию диетолога и психолога/психотерапевта в НМИЦ эндокринологии с явкой на консультацию для оценки клинического эффекта и с результатами УЗИ малого таза через 3 мес.

В течение 4 лет пациентка ежегодно динамически наблюдалась в НМИЦ эндокринологии, продолжала принимать МГТ — эстрадиол/дидрогестерон 1/10. В последний год отметила отсутствие регулярной МПР реакции.

В 52 года на ежегодной плановой консультации пациентка переведена на эстрадиол/дидрогестерон 1/5 и продолжила ежегодное наблюдение.

В январе 2025 г. пациентка Л., 67 лет, отметила отсутствие жалоб и желание пройти ежегодное обследование на фоне приема МГТ (эстрадиол/дидрогестерон 0,5/2,5).

Менопаузальная гормональная терапия (МГТ) является частью единой стратегии поддержания качества жизни и здоровья женщин в пери- и ранней менопаузе. Согласно клиническим рекомендациям МЗ РФ в настоящее время в России зарегистрированы следующие комбинации эстрогенов и гестагенов для терапии климактерических расстройств у женщин с интактной маткой: комбинации для циклического приема: эстрадиол валерат 2 мг + левоноргестрел 0,15 мг; эстрадиол 2 мг или 1 мг + дидрогестерон 10 мг; эстрадиола валерат 2 мг + ципротерон ацетат 2 мг; комбинации для непрерывного приема: эстрадиол 1 мг или 0,5 мг + дидрогестерон 5 мг или 2,5 мг соответственно; эстрадиол 1 мг или 0,5 мг + дроспиренон 2 мг или 0,25 мг соответственно.

## ОБСУЖДЕНИЕ

Согласно данным мировой статистики, ярко прослеживающимися демографическими тенденциями в настоящее время являются увеличение продолжительности жизни и общее старение населения [2]. Эти изменения ведут ко все большему увеличению доли женщин, находящихся в периоде постменопаузы, у 85% из которых менопауза сопровождается развитием патологических состояний, таких как вазомоторные симптомы, психоэмоциональные нарушения и урогенитальные расстройства. В этот период также возможно возникновение долгосрочных рисков для здоровья: переломов шейки бедра вследствие остеопороза, развития сердечно-сосудистых заболеваний, сахарного диабета и т. д. [2][6]. Общепризнано, что менопаузальная гормональная терапия (МГТ) — наиболее эффективный и патогенетически обоснованный метод коррекции климактерических расстройств, являющийся основой поддержания здоровья женщин в пери- и постменопаузе наряду с обязательным соблюдением здорового образа жизни [5][10][11]. В научных исследованиях показано, что МГТ может предотвратить вазомоторные симптомы в 75% случаев, снизить риски перелома шейки бедра на 30%, случаи развития сахарного диабета — на 30%; сердечно-сосудистую смертность — на 12–54%, а также дополнительно может снизить общую смертность на 31% у женщин в возрасте 50–59 лет [2][12]. В настоящее время применение МГТ в Российской Федерации (РФ) в доле от числа женщин в возрасте от 45 до 69 лет составляет 1,3%, что в 2,5 раза ниже, чем в странах Европейского Союза, и примерно в 5 раз ниже реальной потребности населения РФ. Экономический эффект, посчитанный через количество предотвращенных дней нетрудоспособности и сохраненных жизней, составляет 9,1 млрд руб. в год. Если МГТ в РФ будет принимать хотя бы такая же доля женщин, как и в развитых странах, медико-демографический и экономический эффекты возрастут в 2,5 раза по сравнению с настоящим уровнем и составят до 15,4 млрд руб. [2][6].

Для женщин, не нуждающихся в контрацепции, в перименопаузе рекомендовано стартовать с циклической низкодозированной терапии: например, 1 мг эстрадиола/10 мг дидрогестерона. Эффект лечения оценивают индивидуально через 1–2 месяца, и в случае сохранения симптомов возможно перейти на стандартную дозу комбинированного препарата, содержащего 2 мг эстрадиола. После старта и применения циклической комбинированной МГТ женщинам может потребоваться переход на непрерывную комбинированную МГТ в следующих ситуациях: через 1–2 года приема низкодозированной МГТ в циклическом режиме при возрасте женщины на момент начала терапии старше 50 лет; по достижении пациенткой среднего возраста менопаузы (51–52 года), при начале использования МГТ до 50 лет; при изменении характера менструалоподобной реакции: скудные мажущие выделения/полное отсутствие в течение 2 последовательных циклов.

Женщинам в постменопаузе (≥12 месяцев после последней менструации) рекомендуется использовать комбинированную МГТ в монофазном режиме и стартовать с низкодозированного препарата, содержащего в качестве эстрогенного компонента 1 мг эстрадиола (например, эстрадиол 1 мг/дидрогестерон 5 мг). На фоне МГТ проводятся ежегодное обследование пациентки и оценка польза/риск продолжения лечения.

Через 3–5 лет приема низкодозированной комбинированной МГТ в монофазном режиме возможен переход на ультранизкодозированный препарат (например, эстрадиол 0,5 мг/дидрогестерон 2,5 мг) [7][8].

С целью регистрации воспроизведенных препаратов проводятся клинические исследования их биоэквивалетности или терапевтической эквивалентности референтному (как правило, оригинальному) препарату [9].

Было проведено открытое рандомизированное перекрестное двухпериодное исследование биоэквивалентности препаратов PZT-02/2023 (Дидрогестерон) (ООО «Фармасинтез-Тюмень», Россия) и препарата сравнения (Дюфастон®, таблетки, покрытые пленочной оболочкой, 10 мг (Эбботт Биолоджикалз Б.В., Нидерланды/АО «ВЕРОФАРМ», Россия)) у здоровых субъектов женского пола в постменопаузе после однократного приема каждого из препаратов натощак. На основании полученных данных было показано, что исследуемые препараты характеризуются высокой степенью сходства показателей фармакокинетики.

Индивидуальные и усредненные профили фармакокинетических кривых дидрогестерона тестируемого и референтного препаратов имеют совпадающие формы. Препараты характеризуются близкими значениями относительной биодоступности и максимальной концентрации дидрогестерона. 90%-ные доверительные интервалы для отношений средних геометрических значений AUC0-t и Cmax дидрогестерона тестируемого и референтного препаратов в исходных единицах полностью соответствуют допустимым пределам 80,00–125,00% (рис. 1).

**Figure:**
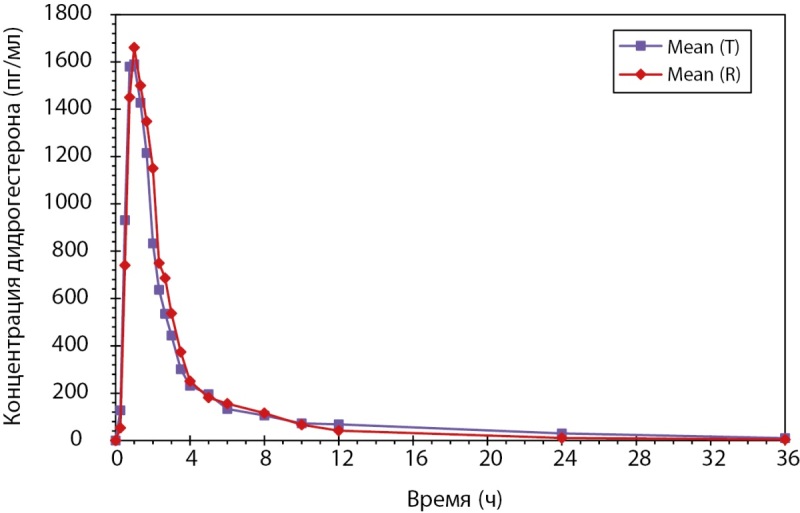
Рисунок 1. Усредненные (среднее арифметическое) фармакокинетические профили концентрации дидрогестерона в плазме крови субъектов после однократного приема тестируемого и референтного препаратов в линейных координатах.

Таким образом, по результатам клинического исследования биоэквивалетности был сделан вывод, что воспроизведенный препарат PZT-02/2023 (дидрогестерон), таблетки, покрытые пленочной оболочкой 10 мг (ООО «Фармасинтез-Тюмень», Россия), и референтный препарат Дюфастон®, таблетки, покрытые пленочной оболочкой 10 мг (Эбботт Хелскеа Продактс Б.В., Нидерланды), являются биоэквивалентными. В ходе данного клинического исследования не было зарегистрировано нежелательных явлений (НЯ) и сделан вывод о сопоставимом профиле безопасности исследуемых препаратов, который можно охарактеризовать как хороший.

Было проведено открытое рандомизированное перекрестное двухпериодное исследование биоэквивалентности препаратов PZT-03/2023 (дидрогестерон + эстрадиол), таблетки, покрытые пленочной оболочкой 2,5 мг + 0,5 мг (ООО «Фармасинтез-Тюмень», Россия), и Фемостон® мини, таблетки, покрытые пленочной оболочкой 2,5 мг + 0,5 мг (Эбботт Хелскеа Продактс Б.В., Нидерланды), у здоровых субъектов женского пола в постменопаузе после однократного приема каждого из препаратов натощак. По результатам данного исследования был сделан вывод, что препараты PZT-03/2023 (Дидрогестерон + Эстрадиол), таблетки, покрытые пленочной оболочкой 2,5 мг + 0,5 мг (ООО «Фармасинтез-Тюмень», Россия), и Фемостон® мини, таблетки, покрытые пленочной оболочкой 2,5 мг + 0,5 мг (Эбботт Хелскеа Продактс Б.В., Нидерланды) являются биоэквивалентными (рис. 2 и рис. 3). Исследование также показало, что препараты обладают схожим профилем безопасности.

**Figure:**
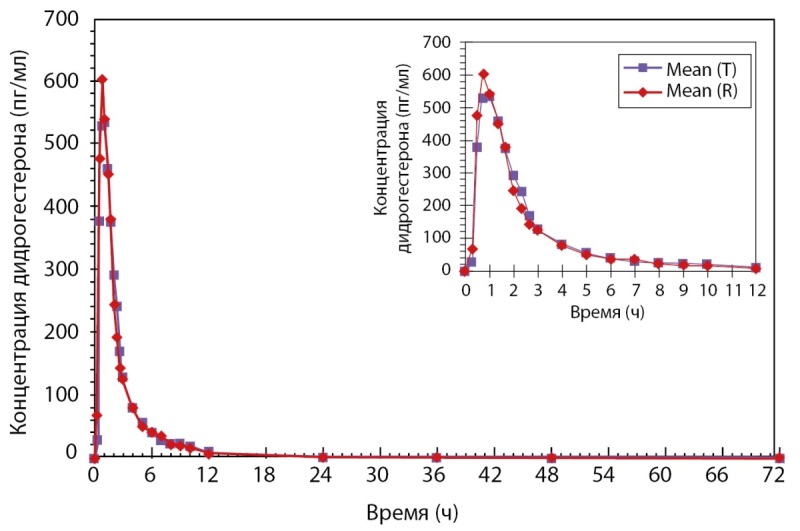
Рисунок 2. Усредненные (среднее арифметическое) фармакокинетические профили концентрации дидрогестерона в плазме крови субъектов после однократного приема тестируемого и референтного препаратов в линейных координатах.

**Figure:**
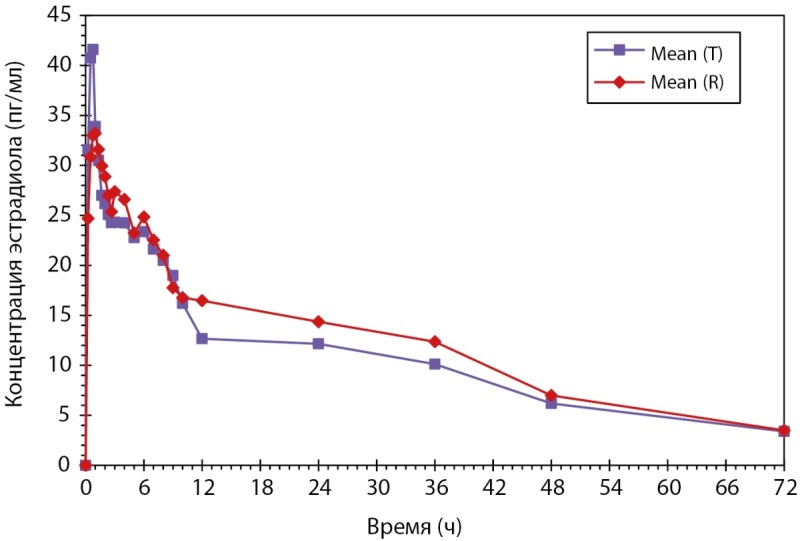
Рисунок 3. Усредненные (среднее арифметическое) фармакокинетические профили концентрации эстрадиола в плазме крови субъектов после однократного приема тестируемого и референтного препаратов в линейных координатах.

Было проведено открытое рандомизированное перекрестное двухпериодное исследование биоэквивалентности препаратов PZT-04/2023 (Дидрогестерон + Эстрадиол), таблетки, покрытые пленочной оболочкой 5 мг + 1 мг (ООО «Фармасинтез-Тюмень», Россия), и Фемостон® конти, таблетки, покрытые пленочной оболочкой 5 мг + 1 мг (Эбботт Хелскеа Продактс Б.В., Нидерланды), у здоровых субъектов женского пола в постменопаузе после однократного приема каждого из препаратов натощак. По результатам данного исследования был сделан вывод: препараты PZT-04/2023 (Дидрогестерон+Эстрадиол), таблетки, покрытые пленочной оболочкой 5 мг + 1 мг (ООО «Фармасинтез-Тюмень», Россия), таблетки, покрытые пленочной оболочкой 10 мг (ООО «Фармасинтез-Тюмень», Россия), и Фемостон® конти, таблетки, покрытые пленочной оболочкой 5 мг + 1 мг (Эбботт Хелскеа Продактс Б.В., Нидерланды), являются биоэквивалентными (рис. 4 и рис. 5). Исследование также показало, что препараты обладают схожим профилем безопасности.

**Figure:**
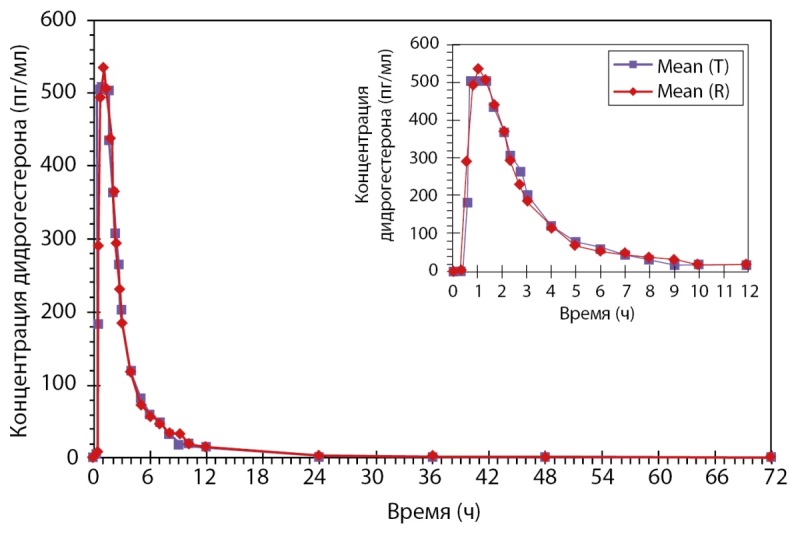
Рисунок 4. Усредненные (среднее арифметическое) фармакокинетические профили концентрации дидрогестерона в плазме крови субъектов после однократного приема тестируемого и референтного препаратов в линейных координатах.

**Figure:**
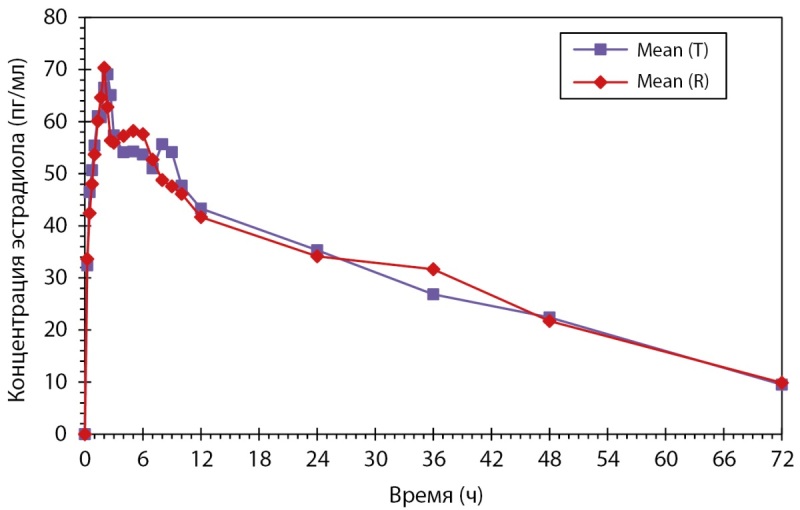
Рисунок 5. Усредненные (среднее арифметическое) фармакокинетические профили концентрации эстрадиола в плазме крови субъектов после однократного приема тестируемого и референтного препаратов в линейных координатах.

Было проведено открытое рандомизированное перекрестное четырехпериодное исследование биоэквивалентности препаратов PZT-22/2023 (Дидрогестерон+Эстрадиол и Эстрадиол [набор]) (ООО «Фармасинтез-Тюмень», Россия) и препарата сравнения у здоровых субъектов женского пола в постменопаузе после однократного приема каждого из препаратов натощак. По результатам данного исследования был сделан вывод, что препараты PZT-22/2023 (Дидрогестерон + Эстрадиол и Эстрадиол [набор]), набор таблеток, покрытых пленочной оболочкой, 10 мг + 2 мг и 2 мг (ООО «Фармасинтез-Тюмень», Россия), и Фемостон® 2, набор таблеток, покрытых пленочной оболочкой, 10 мг + 2 мг и 2 мг (Эбботт Хелскеа Продактс Б.В., Нидерланды), являются биоэквивалентными (рис. 6 и рис. 7), а также обладают схожим профилем безопасности.

**Figure:**
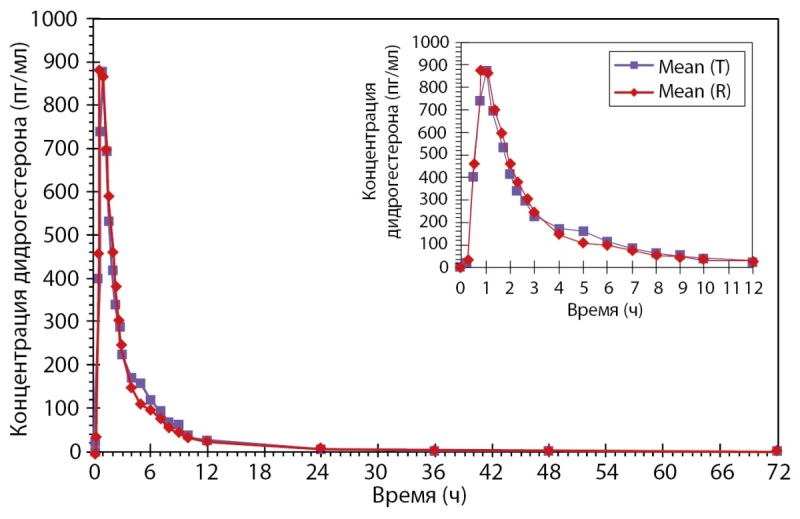
Рисунок 6. Усредненные (среднее арифметическое) фармакокинетические профили концентрации дидрогестерона в плазме крови субъектов после однократного приема тестируемого и референтного препаратов в линейных координатах в 1 и 2 периоде.

**Figure:**
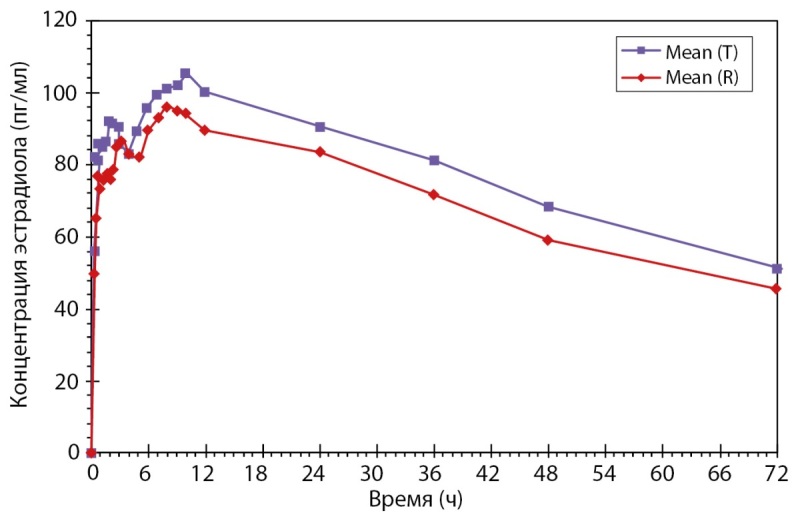
Рисунок 7. Усредненные (среднее арифметическое) фармакокинетические профили концентрации эстрадиола в плазме крови субъектов после однократного приема тестируемого и референтного препаратов в линейных координатах в 1 и 2 периодах.

Все упомянутые выше исследования были проведены в соответствии с Протоколом клинического исследования и соответствующими требованиями российского законодательства, и международными правилами проведения клинических исследований (ICH GCP). По результатам исследования можно сделать вывод о схожем профиле безопасности исследуемых препаратов. Согласно заключению по безопасности, переносимость препаратов была хорошей.

## ЗАКЛЮЧЕНИЕ

Своевременная и индивидуально подобранная по показаниям менопаузальная гормональная терапия (МГТ) является ключевым методом поддержания здоровья и качества жизни женщин, а также профилактики заболеваний, ассоциированных с менопаузой. У соматически здоровых женщин польза применения МГТ перевешивает возможные риски, но ситуация усложняется и требует особого внимания специалистов у коморбидных пациенток. При этом новым ожиданием человечества на сегодняшний день является активная старость. МГТ должна подбираться с учетом выраженности симптомов менопаузы, наличия сопутствующих заболеваний, переносимости препаратов и других персональных особенностей пациентки; также важно учитывать доступность в стране назначаемых лекарственных средств для длительного применения. Правильный алгоритм действий врача во многом будет обеспечивать эффективность проводимого лечения, безопасность, а также приверженность женщины к назначаемой терапии.

Среди препаратов, предназначенных для коррекции менопаузальных симптомов, комбинация эстрадиол/дидрогестерон соответствует всем критериям МГТ, согласно данным Клинических рекомендаций МЗ РФ «Менопауза и климактерическое состояние», а также международных обществ по менопаузе (IMS, EMAS, NICE). Линейка различных дозировок и режимов комбинации эстрадиол/дидрогестерон позволяет максимально персонализировать терапию климактерических расстройств и обеспечивает возможность ведения пациентки на протяжении всего периода — от пери- до постменопаузы, не меняя гестаген.

## ДОПОЛНИТЕЛЬНАЯ ИНФОРМАЦИЯ

Источники финансирования. Работа выполнена с привлечением материалов клинических исследований по биоэквивалентности компании ООО «Фармасинтез-Тюмень».

Конфликт интересов. Авторы декларируют отсутствие явных и потенциальных конфликтов интересов, связанных с содержанием настоящей статьи.

Участие авторов. Все авторы одобрили финальную версию статьи перед публикацией, выразили согласие нести ответственность за все аспекты работы, подразумевающую надлежащее изучение и решение вопросов, связанных с точностью или добросовестностью любой части работы.
